# Early evolution of efficient enzymes and genome organization

**DOI:** 10.1186/1745-6150-7-38

**Published:** 2012-10-31

**Authors:** András Szilágyi, Ádám Kun, Eörs Szathmáry

**Affiliations:** 1 Department of Plant Taxonomy and Ecology, Institute of Biology, Eötvös University, Pázmány Péter sétány 1/C, 1117, Budapest, Hungary; 2 Department of Plant Taxonomy and Ecology, Research Group of Ecology and Theoretical Biology, Eötvös University and The Hungarian Academy of Sciences, Pázmány P. sétány 1/C, H-1117, Budapest, Hungary; 3 Parmenides Center for the Conceptual Foundations of Science, Kirchplatz 1, D-82049, Munich/Pullach, Germany

**Keywords:** Origin of life, Chromosome, Metabolism, Ribozyme, Major transitions, Enzyme evolution

## Abstract

**Background:**

Cellular life with complex metabolism probably evolved during the reign of RNA, when it served as both information carrier and enzyme. Jensen proposed that enzymes of primordial cells possessed broad specificities: they were generalist. When and under what conditions could primordial metabolism run by generalist enzymes evolve to contemporary-type metabolism run by specific enzymes?

**Results:**

Here we show by numerical simulation of an enzyme-catalyzed reaction chain that specialist enzymes spread after the invention of the chromosome because protocells harbouring unlinked genes maintain largely non-specific enzymes to reduce their assortment load. When genes are linked on chromosomes, high enzyme specificity evolves because it increases biomass production, also by reducing taxation by side reactions.

**Conclusion:**

The constitution of the genetic system has a profound influence on the limits of metabolic efficiency. The major evolutionary transition to chromosomes is thus proven to be a prerequisite for a complex metabolism. Furthermore, the appearance of specific enzymes opens the door for the evolution of their regulation.

**Reviewers:**

This article was reviewed by Sándor Pongor, Gáspár Jékely, and Rob Knight.

## Background

The major evolutionary transitions [1] set a timeline onto which other evolutionary milestones can be integrated. The emergence of complex metabolism in the RNA world [2-4] (an age when RNA served as both information carrier and enzyme) is one such milestone, whose place in the order of events has not yet been determined. Some rudimentary metabolism could have existed on mineral surfaces [5], where RNA oligomers can also form [6]. Template-based replication of these oligomers was achieved at this stage, which transformed RNA molecules into units of evolution. These independent replicators became compartmentalized during the first major evolutionary transition [1], and by their very nature, possessed at least the ability to enhance their own formation. A good fraction of early ribozymes (RNA enzymes) was likely to have been inefficient generalists [7], as it must have taken time to optimize their function. Furthermore, the ever-changing and unpredictable primordial environment probably favored broad specificities and the ability to adapt to new substrates [8]. By the invention of translated protein synthesis [1], a complex metabolism was likely in place. We can conclude that a metabolism driven by specialist enzymes is likely to have emerged in the RNA world [2], before the invention of the genetic code and translation.

Evolution of complex metabolism requires that enzymes be able to evolve from one function to another; and be able to reach high rate enhancement and specificity. The plethora of artificially evolved ribozymes [3,4,9] testify that RNA is well capable of acquiring novel catalytic functions. Furthermore, evolution can lead from one enzyme function to another (e.g. the Bartel I ligase that was turned into an RNA polymerase [10,11]). RNA enzymes are capable of very specific catalysis with potentially high catalytic rate enchantment [12]. Thus there is no biochemical reason for not having specific enzymes rather soon after the appearance of ribozymes. The possibility of division of labor and evolution of specialist enzymes has also been demonstrated in theoretical studies of surface metabolism [13] and compartmentalized systems [14], however only for a few enzyme specificities and without modeling of enzyme-substrate interactions. Another theoretical investigation, however, found limited evolution towards specialist enzymes [15].

The question we address in this paper is whether specialist enzymes evolved before or after the establishment of chromosomes.

## Methods

**Figure 1:**
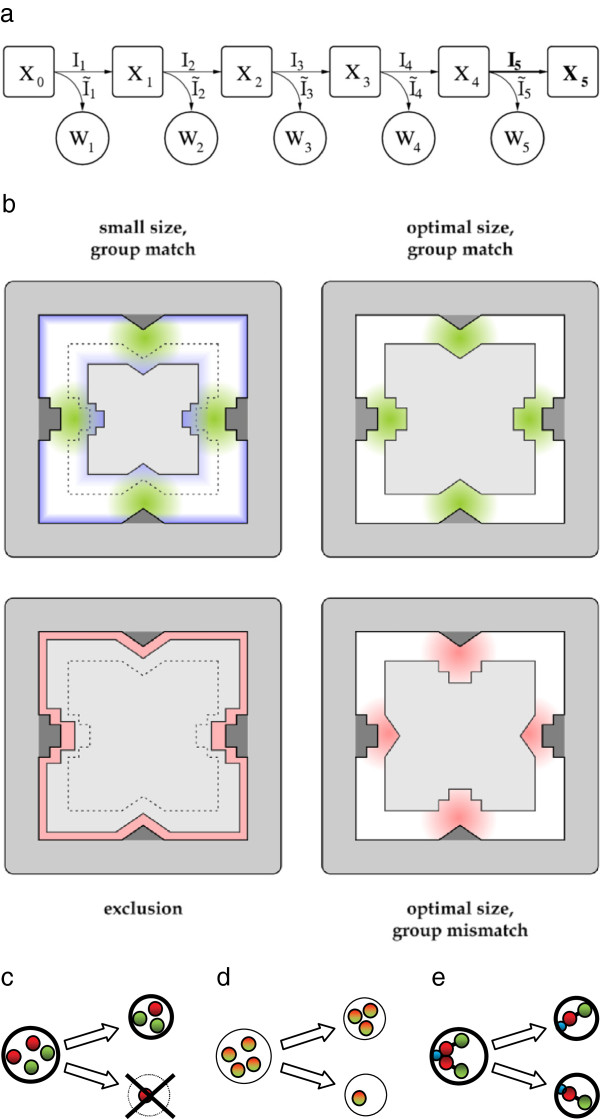
**a. Reaction scheme.** X’s represent the products, W’s the waste products. The final flux (in this five step reaction *I*_5_) is the fitness of a given cell. **b.** Relative orientation of enzyme-substrate complex. If the functional groups totally match and the distance between the faces (*R*_*i*_) are at the optimal value of the van der Waals interaction (*R*_opt_, marked by dashed line) the conversion has the highest activity (*top right*). If the enzyme is larger the conversion yield reduces (*top left*), if the enzyme is too small the substrate is sterically excluded (*bottom left*). If the functional groups differ in two functional groups the enzyme catalyses waste production (*bottom right*). **c.** independently replicating genes and random assortment to daughter cells (specialist enzymes in green and red); **d.** generalist enzymes; and **e.** protocell with a chromosome and accurate segregation mechanism attached to cell boundary (blue). Fitter protocells are marked by thicker boundaries.

We follow the evolution of enzyme specificities acting on a linear series of reactions (Figure 1a) with a model similar to that used in the pioneering study of Kacser and Beeby [15]. Substrates and enzymes are represented by hyper-blocks and cavities of *D* dimensions, and both have functional groups at each face (an important deviation from the cited model) (Figure 1b). The fit between the substrate and the enzyme determines catalytic activity, while the match between the functional groups determines whether the reaction produces a component in the biomass producing line of reaction (Figure 1b, top), a waste product (Figure 1b, bottom right) or nothing (Figure 1b, bottom left). More specifically, the enzymatic activity is calculated from the binding energy of the enzyme–substrate complex using the Lennard-Jones equation: $\varepsilon =2\sum_{j=1}^{D}\left(1/R_{j}^{12}-10/R_{j}^{6}\right)$, where *R*_*j*_ is the distance between the wall of the cavity and the face of the centered substrate (see Figure 1b). We assume that the catalytic activity is proportional to this energy: *e* = − *ε* (thermodynamics would require the natural logarithm of this, but since the function remains monotonous we neglected it for speeding up the rate of evolution, thus reducing simulation time). Each reaction step *i* (both forward and waste) is treated as a simplified Michaelis–Menten step with unsaturated enzymes, thus the flux toward biomass accumulation is *I*_*i*_ = *e*_*i*_(*X*_*i* − 1_ − *X*_*i*_) and the flux toward waste is *Ĩ*_*i*_ = 0.5*e*_*i*_*X*_*i* − 1_. We further assume that *X*_0_ = 1, *X*_final_ = 0 and *W*_*i*_ ≈ 0 (assuming that waste products diffuse quickly, and it cannot be converted back to non-waste product). Coupled with the equations ensuring conservation of flux (*I*_*i*_ = *I*_*i* + 1_ + *Ĩ*_*i* + 1_), any of the fluxes can be computed. The flux of the last catalytic reaction (*I*_final_) determines the rate of biomass accumulation, which in turn translates to rates of protocell division (upon reaching a certain number of ribozymes or total flux). As this flux determines the replication rate of the protocell, it serves as a measure of fitness. Detailed derivation of the model is found in the Appendix.

The population dynamics of the protocell follows a Moran process, i.e. when a protocell divides one of the daughter cells replaces the original protocell, and the other replaces a randomly chosen protocell from the population. By using this update rule we assume that the population size is constant (*N* = 5000). Protocells divide when the *cumulative* flux of the system reaches a threshold (*C*_*I*_^crit^ = 100). Upon replication of the genes mutations can occur in the genes of the protocell. Each sides of the enzyme is altered by a random number obtained from a normal distribution with mean 0 and *σ* = 0.05 standard deviation.

We implemented three separate versions of the model: in one (version 1), ribozymes replicate individually, in the second (version 2) chromosomes sometimes form, but genes mostly replicate individually; and in the third (version 3), genes are permanently linked together in a chromosome. In the presence of a chromosome the *cumulative* flux of the system needs to reach a threshold in order for the protocell to divide (see above). In the individually replicating ribozymes case (version 1), whenever the cumulative flux exceeds a value (*C*_*I*_^dd^ = 6.7) a new replicator is added to the protocell till the number of independent replicators reaches the threshold (*n*^crit^ = 15) value. The protocells divide at the same cumulative flux as in the chromosome case, as *C*_*I*_^crit^ = *n*^crit^ ⋅ *C*_*I*_^add^. In version 2, the total number of genes need to reach the threshold is *n*^crit^ = 15, irrespective of them being individually present or linked into a chromosome. Here, independent ribozymes replicate if the cumulative flux exceeds the value *C*_*I*_^add^ = 6.7, and chromosomes replicate if the cumulative flux exceeds the same value times the number of genes in the chromosome. The new replicator is produced by copying and mutating a randomly chosen ribozyme present within the protocell. In the 2^nd^ version of the model there is a 10^-3^ chance at each time step that the genes form a chromosome, and will replicate together from that point of time. At cell division, the genetic materials are divided among the daughter protocells. Either both of them gain one copy of the chromosome (version 3), or each ribozyme (version 1 and 2) or chromosome (version 2) is randomly assigned to one of the daughter protocells.

Initially all protocells are identical, and all ribozymes are totally generalist (as a worst-case assumption), i.e. they are large enough to fit onto every substrate. In the 1^st^ version of the model, the protocells initially harbor as many ribozymes as there are reactions. In the 2^nd^ version of the model, all genes start as individually replicating and there are no chromosomes in any of them. We followed the evolutionary dynamics till equilibrium.

## Results and discussion

The three versions of the model represent three stages of chromosome evolution. The initial phase of no chromosomes (version 1), the transitional stage when genes can link up to chromosomes, but assortment to daughter cells is still random (version 2), and the final stage of a fully formed chromosome with exact mechanism of distributing one copy to each daughter cell (version 3).

**Figure 2:**
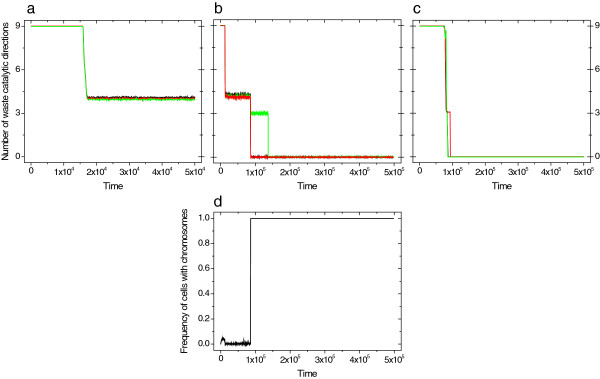
**Number of waste catalytic direction in a 3-step reaction network as a function of time in protocells with (a) individually replicating genes and random assortment; (b) potential for chromosome formation and random assortment; and (c) in protocells with chromosomes.** Each line (black, red and green) shows the number of waste catalytic directions of the enzymes. For combinatorial and geometrical reasons the maximum number of waste catalytic direction in 3 dimensions is 9. (**d**) Change of frequency of cells with chromosome through time. The dynamic of the population is followed for 10^6^ time steps, and the first 5·10^5^ or 5·10^4^ is shown with data recorded at every 10^3^ time steps. The sizes of the three substrates are given by cyclic permutation of the sizes 1.0, 2.5 and 4.0 (1.0×2.5×4.0, 2.5×4.0×1.0 and 4.0×1.0×2.5). Initially all enzymes has size of 7.0×7.0×7.0, which is large enough to fit onto every substrate.

We get markedly different results for the three versions of our model, which only differ in how genes are assorted into daughter cells. Specialized enzymes do not evolve in protocells with individually replicating genes and random assortment (no chromosome case) even if there are only three reactions to catalyze (Figure 2a). Ribozymes remain generalist and a considerable number of side reactions can be observed. In contrast, protocells with chromosomes evolve toward fully specialist enzymes that efficiently channel flux toward biomass accumulation (Figure 2c). The inability of individually segregating replicators to become fully specialized rests on the high probability of losing a gene at protocell division (the assortment load). Protocells with less specific enzymes reduce the assortment load by maintaining metabolism at moderate efficiency. There is an intermediate evolutionary stage connecting these two cases, as demonstrated by the results of the 2nd version of our model. Here the system first evolves to the specificity exhibited in the no chromosome case (Figure 2b). Only then could chromosomes form, because a newly formed chromosome can decrease the assortment load and give rise to a fully specialized system (compare Figure 2b and c). It is worth mentioning that in the intermediate system there is still some assortment load, as the chromosomes still assort randomly. Thus a beneficial mutation appearing in one chromosome, and helping the cell to divide faster might not get into one of the daughter cells. This, however, does not affect the end result, which is full specialization.

**Figure 3:**
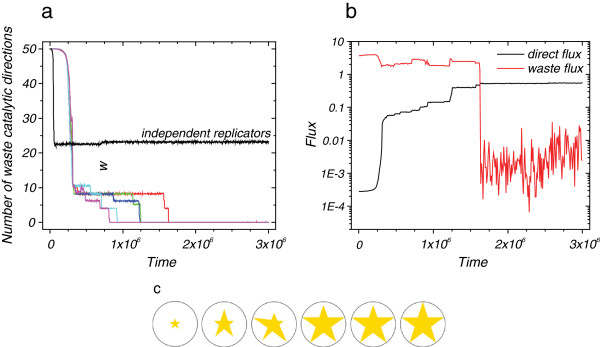
**Evolution of (a) the number of waste catalytic directions (color lines correspond to the population average of the five enzymes of the system with chromosomes; black line shows the average number of waste catalytic directions in case of independent replicators) and (b) the flux (both direct and waste) in the 5-step reaction network.** Both fluxes are normalized (the unity of the direct flux corresponds to the optimal enzyme configuration). The direct flux fails to reach its maximal value due to recurrent mutations. (**c**) The stars represent the catalytic activities of the five enzymes relative to the maximal value represented by the circle at every 5·10^5^ time step, beginning at 2·10^5^. The sizes of the five substrates are 1.0×2.5×4.0×5.5×7.0, and the cyclic permutation of these sizes. Initially all enzymes is a hypercube with a side of 9.0. These cubes are large enough to fit onto every substrate.

The invention of the chromosome allowed many specialized enzymes to evolve from initially generalist enzymes. We demonstrate that specific enzymes also evolve for longer reaction chains (Figure 3). Here a reaction chain consisting of five reactions is considered. Evolution quickly eliminates most of the catalysis toward unproductive side reactions, but roughly nine waste directions remain in the system for a long time (Figure 3a). From here, enzyme evolution slows down considerably, although in the end all enzymes find the optimal shape (Figure 3a). In the beginning the flux toward unproductive side reactions is nearly 4 orders of magnitude larger than toward biomass, thus there is a strong selection for preventing unproductive side reactions. As a result, the flux toward biomass increases by 3 orders of magnitude, although flux toward the waste decreases only slightly (Figure 3b). The enzymes evolve to a shape that excludes most of the side reactions (hence the increase in flux toward biomass), but at the same time increases activity toward a few of them (hence the only slight decrease in flux toward waste). A marked drop in the flux toward waste can only be observed when the last of the waste directions is eliminated. In the end, flux toward biomass accumulation increases by four magnitudes, while flux toward the waste directions decerases by three magnitudes resulting in a ca. 10^7^ enhancement in specificity.

The longer the reaction chain the longer it takes evolution to find the optimal solution (compare the time scales in Figure 2c and Figure 3a), but the solution is always found. For this reason the above observation can be extended to arbitrary reaction chain length (we have also obtained results for chain length of 6, data not shown) and different topologies, as there is nothing to suggest that the same mechanism could not work for longer reaction sets and more complex networks. However, longer reaction chains are computationally more demanding, and it quickly becomes unfeasible to follow as the number of reaction steps increases.

Our results are robust to the details of the model: changing the mutational variance or the redundancy of ribozymes within the protocells, or the introduction of fluctuating inflow of starting material, do not change our results in a qualitative manner.

**Figure 4:**
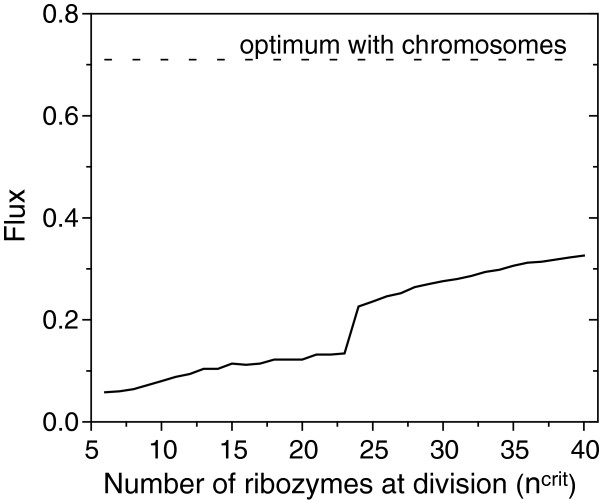
**The normalized flux at equilibrium as the function of the number of enzymes at protocell division (*n***^***crit***^**).** Other parameters as in Figure 2.

Assortment load can be lowered by increasing the internal copy number of replicators (*n*^crit^), although it also entails fitness costs (in terms of energy and speed). There is an increase of the attainable equilibrium flux of the last catalytic step by more efficient reduction of catalytic enhancement toward waste directions with the increase of *n*^crit^; the increase is very modest (Figure 4). While assortment load is known to vanish at *n*^crit^ → *∞*[16], however, this ideal state is approached very slowly, and for realistic numbers of internal molecules the attainable final flux remains well below the optimal flux observed in protocells with a chromosome. We can thus conclude that within a reasonable range of *n*^crit^ values, our qualitative result holds: full specialization could not be reached even if each replicator was in a great excess (Figure 4).

**Figure 5:**
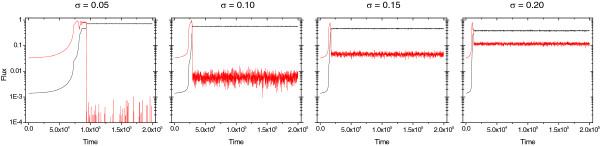
**The evolution of mean flux (both direct and waste) in a 3-step reaction network.** Both fluxes were normalized (the unity corresponds to the optimal enzyme configuration). Mean flux is determined every 10^3^ time steps. The model was run for 5·10^5^ time steps, results for the first 2·10^5^ time steps are shown. All replicate populations reached the fully specialized state in equilibrium. Other parameters are as in Figure 2.

Our choice of mutational variance, *σ* = 0.05, allows convergence to equilibrium in reasonable time and at the same time it keeps fluctuation due to stochasticity down. Smaller mutational variance increases the time it takes to reach equilibrium and the final flux tends to its optimal value (unity), while a higher mutational variance decreases the mean flux toward biomass accumulation in a fully specialized population (Figure 5). Thus, our choice of mutational variance does not affect our qualitative outcomes.

**Figure 6:**
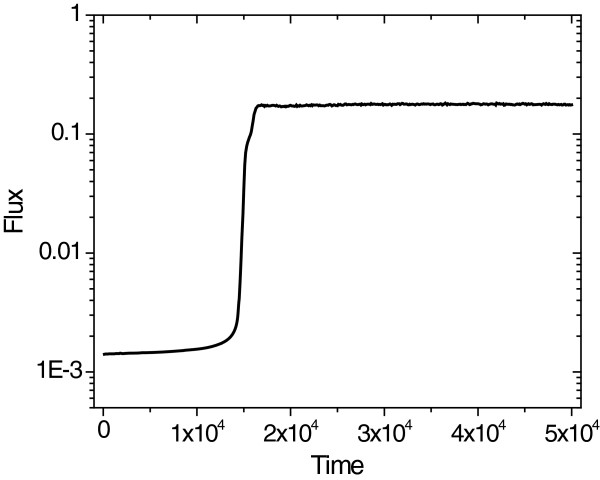
**The evolution of mean direct flux in the 3-step reaction network with individually replicating genes.** Other parameters are as in Figure 2.

We have checked the case when catalytic activity was proportional to the natural logarithm of the binding energy: *e* = − ln *ε*, as dictated by thermodynamics. Results for reaction chain length of 3 show qualitatively the same results as results without the logarithm (data not shown).

We conclude that our results are robust, and the same qualitative outcome can be observed with modified versions of the model and/or in a vast area of the parameter space. Accordingly, it is important to understand why Kacser and Beeby have not achieved the stage of nearly complete enzyme specificity [17,18], despite assuming that genes sit on chromosomes. There are three crucial differences: we count with more than 3 dimensions for enzyme-substrate fit increasing the potential of full speciality; we consider functional group identity; and as a consequence we allow for harmful side reactions. They calculated with active centre boxes only: it is easy to see that in three dimensions one cannot evolve fully specific enzymes for a linear pathway of 8 reactions. It is thus not surprising that they found mere partitioning of catalytic task space (*sensu* Kauffman [19]) without attaining high specificity. Furthermore, this partitioning allowed for historically contingent end states, which they indeed found to happen.

Our results suggest that chromosome formation preceded complex metabolism run by specific enzymes, but they do not suggest that no specific enzyme could form. We have set each of our reactions equally important, but none needed to be specific in order to function (albeit higher specificity bestowed a fitness advantage). The system with independently replicating genes evolved to a stage in which the opposing selective forces favoring fewer genes because of the assortment load, and higher efficiency due to specificity cancelled each other out. However, we know that specific enzymes (i.e. two enzymes that are both required for a functional cell) can coexist despite the assortment load [20]. Certain cellular functions might require highly efficient and/or specific enzymes. The two are not necessary the same. For example, a replicase needs to be efficient (see below), but should at the same time be a generalist in the sense that it should be able to replicate any sequence. We hypothesize that a few specific and a larger number of generalist enzymes could have coexisted before the evolution of the chromosome.

Linkage of genes and complexity and specificity of metabolism coevolved. Maynard Smith and Szathmáry have demonstrated that the chromosome can evolve by genes linking together and outcompeting the cells with independently replicating genes [21], which we have also shown. In our model, linkage went to fixation only after specificity reached the level attainable in a system with independently replicating genes, even though chromosome-harboring cells appeared earlier, but these were competed out. Thus we show that genetic representation and metabolism coevolve. Our simple model cannot capture all the necessary ingredients of the evolution of the chromosome, for example the extra enzymatic functions required [22]. Two novel functions need to evolve: an RNA endonuclease enzyme that liberates the ribozymes from the chromosome, and some way to attach the chromosome to the cell boundary, so that the growth of the boundary can help separate the two copies. The first enzymatic function is straightforward: all extant ribozymes cleave RNA [23] and the simple structural motifs exhibited by the hairpin or the hammerhead ribozymes are common even in random pools of short RNAs [24]. Moreover, an enzyme that can cleave RNA is often also proficient in ligating them, a function which is essential for the formation of the chromosome, although chromosomes could have emerged by recombination as well. For the second function, chromosome separation, something that attached the chromosome to the cell wall is required (assuming that the cell has a cell wall, like most prokaryotes do) [22]. This linkage could be a small peptide.

## Conclusion

Our results demonstrate that a highly specific enzyme set is unlikely to evolve before the invention of chromosomes. The appearance of chromosomes is made possible by considerable increase in the fidelity of replication, as the amount of the genetic information, and thus the number of different enzymes a protocell could have had, is limited by the fidelity of the copying process [25]. For example, the 99.4% copying fidelity exhibited by the putative replicase ribozyme [10] would allow for a genome having roughly 1,200 nucleotides [26], still nearly a magnitude less than estimated for a minimal ribo-organism [26]. In order to overcome this error threshold the genetic information needs to be maintained as individual replicators [20,27]. However, when replicators replicate individually then there is intragenomic conflict [25], as the fastest replicator tends to dominate the system, thereby causing the loss of other replicators, and thus information. This internal conflict can be suppressed in a small, randomly assorted population of compartmentalized replicators, where the stochastic nature of segregation to daughter protocells upon division can, through the generation of a more equally distributed gene set, ensure the maintenance of the full diversity of the original set of enzymes [20]. However, random assortment sets another error threshold: the number of different replicators that can be maintained is limited by the total number of replicators present. The fidelity of the replication process as well as the control mechanisms that guide the segregation of the chromosome evolved at this stage of the origin of life. Diversified, complex metabolism evolved afterwards.

How diversified and complex the minimal metabolism was is still debated [28,29], but a figure around 200 genes emerges as the minimum for a DNA-peptide organism. This figure, however, contains all the genes for translation and also for DNA replication, functions that did not exist when the chromosome evolved [1]. The minimal gene set suggested by comparison of bacterial genomes [30] includes 95–96 genes for translation, nearly half of the suggested minimal set of 206 genes [30]. Furthermore, there are 15 genes involved in other protein related functions and 16 genes for DNA replication and other DNA related functions (repair, modification, restriction). Thus a ribo-organism could function with less than a 100 genes. The minimal intermediate metabolism is suggested to require 50 enzymes [31]. An RNA-dependent RNA polymerase is required, and if it does not also posses helicase activity, then a separate enzyme for that function is also required (2 genes). We should also include 2 genes for RNA degradation, 1 for cell division, and 4 involved in transport [30]. This gives us an estimate of around 60 genes. Considering that, strictly speaking, this is 60 functions and not 60 genes, the final figure can even be less as generalist enzymes can catalyse more than one of the proposed reactions. This set of enzymes is supposed to be present already at the stage of independent replicators.

A further ingredient of the evolution of increasing enzyme specificity could have been the advantage gained from metabolic regulation. In an unregulated metabolism, cross-catalysis might be neutral or even beneficial (forgetting side reactions for a moment), but if the cell wants to down-regulate enzyme *A* that converts substrate *a,* because the pathway is temporarily not needed, it can easily mean that the conversion rate of some other substrates, say *p* and *z,* will also diminish. *Regulation in general makes sense only with specific targets.* A future goal is simulation of the coevolution of protocell metabolic network and enzymes, using artificial chemistry [32], which in all likelihood will generate further insight into protocell evolution in general, including membrane-metabolism coevolution [33] that may have led from completely heterotrophic protocells [34] to cells with a rich internal metabolic network.

## Competing interests

The authors declare that they have no competing interests.

## Authors’ contributions

All authors participated in the design of the model and the drafting of the manuscript. ASz developed the model and gathered the data. All authors read and approved the final manuscript.

## Reviewers' report

Reviewer 1: Sándor Pongor, International Centre for Genetic Engineering and Biotechnology, Trieste, Italy

Szathmáry and coworkers seek to answer the question re when complex metabolism could have originated in the course of evolution. The question is highly relevant, and to the best of my knowledge it has not been tackled by other studies. Timing in relation to the major evolutionary transitions is an original and elegant idea that is especially suited for modeling studies. The authors propose that specialist enzymes emerged after the appearance of the chromosome because protocells harbouring unlinked genes maintain largely aspecific enzymes to reduce their assortment load.

The authors attack the problem using an elegant model of a population of protocells. The presentation of the model is clear and straightforward, and the authors show that the model is robust in the sense that some changes in the methodology do not affect the qualitative outcome of the simulations. This is where I would like to raise my first question. Metabolism implemented in the paper is based on a linear set of reactions. While there are linear anabolic pathways (e.g. fatty acid synthesis), many of the supposedly ancient pathways have more complex topology. Do the results of the model change if different topologies, in particular the autocatalytic cycle, are also considered?

The underlying equations of fluxes will not qualitatively change if we change the topology of the reaction network. Thus our result will be qualitatively the same for any topology.

As my background is in biochemistry, I cannot resist asking questions regarding the nature of enzyme-substrate interactions. The authors admit that their representation of this interaction is a rather abstract one. I agree that such a representation is adequate for the question at hand. Nevertheless, it should be discussed in some depth, the consequences of the abstraction, in comparison to “real” enzyme-substrate interactions. While Kacser and Beeby employed 3D blocks in their cited study, Szilágyi *et al.* here assume hypercubes of n>3 dimensions. What is the precise meaning of these dimensions? Furthermore, more realistic descriptions, used for instance in classical molecular dynamics, apply a variety of explicitly described molecular interaction types. Why did the authors choose the Lennard-Jones potential, and would it make a difference if other interactions would also be considered?

The active site of an enzyme is a complex cavity, where the relative positions of a number of atoms are key to successful catalysis. Such positioning can only be described by more than 3 values. In reality, the abstract dimensions we employed would translate to distances and angles between side chains/atoms participating in catalysis. In a similar vein any potential function that has one minimum would lead to the same qualitative results as our model, because only the existence of a perfect fit matters here. Thus we could make the model more complex, although it would not alter the qualitative results, but such complexity might blur our clear message by too much technicality.

The results are nicely presented and the underlying mechanism adequately discussed. The mention of regulation in the outlook is very important, there is often much talk about enzyme catalysis, but less about the regulation and coordination required for a truly complex metabolism. That notwithstanding, I missed a discussion of minimal metabolism in the paper. Namely, the metabolic complexity required at different stages of the evolution of life sets a minimum requirement on the number of reactions needed. One would expect that the invention of chromosome would also lead to new enzyme functions. I would like that the authors discuss this matter in the paper.

We now discuss the minimal number of enzymes required for a minimal protocell with and without chromosome.

In summary, I consider this paper will be a welcome addition to the field, and warmly recommend for publication in Biology Direct.

We are grateful for your useful comments, that helped us to improve the manuscript.

Reviewer 2: Rob Knight, University of Colorado Boulder, USA

In this paper, the authors address the question of the relative order in which enzymes with high specificity evolved relative to the evolution of chromosomes, fitting these two events into their "major transitions in evolution" framework. They accomplish this by modeling enzyme evolution according to a block-and-cavity model previously and successfully used for other studies, implemented in two versions: one with ribozymes unlinked, and one with ribozymes linked into chromosomes. Essentially, the model proto-cells either divide once a threshold concentration of the chromosome is reached, or once enough independently replicating RNA enzymes reach sufficiently high concentration (but the daughter cells might not have all the ribozymes). The ribozymes are initially fully general but specialize during the simulation. In the case without chromosomes, the ribozymes remain unspecialized, whereas in the case with chromosomes the ribozymes rapidly specialize to carry out specific reactions. The interpretation is that chromosomes allow specialization because each ribozyme can then guarantee co-occurrence with other, specialized ribozymes.

This work is interesting in that such a clear result, that linkage of functions drives specialization, arises from such a simple, abstract model. I do have some concerns about the generality of the conclusions reached. For example, some other assortment mechanism than chromosomes that would also result in physical partition, for example hybridization of complementary regions or ability to bind a common substrate (e.g. through accessory aptamer domains, or through "zip code"-style packaging signals and apparatus) would be formally equivalent in the model, yet would imply a very different pathway of evolution with respect to chromosomes specifically. Additionally, it might be interesting to explore the implications of linkage for parasitism of the system by non-functional RNAs.

Thank you very much for this comment. As you mentioned our conclusion will not change if other modes of linkage are considered. Once linkage allows the evolution of a more complex metabolism other linkage mechanism could also be explored. Thus, any particular molecular mechanism of linkage suffices, and can give rise to the ligation-based linkage assumed in the chromosome.

The work of Briones et al. doi: 10.1261/rna.1488609 on the evolution of modular RNAs versus single large RNAs is also relevant and should perhaps be discussed.

*Briones et al.*[24]*elegantly demonstrate that the structural diversity of RNA molecules can be extended by the ligation of randomly formed strands. It opens up the possibility of gradual increase in complexity. That study deals with a prior stage in the origin of life, the one leading to a replicase which – we claim – is a prerequisite for the (proto-)cellular stage.*

The equations were missing symbols (notably sigma signs) in the version I reviewed, and this needs to be corrected before publication, along with the language errors noted below.

*We have corrected these errors*.

Overall, I believe this is a valuable contribution to the literature that, with appropriate cautionary notes about the limits of what the model can define, will be of interest to those studying the origins of modern life.

Reviewer 3: Gáspár Jékely, Max Plank Institute for Marine Biology, Tübingen, Germany

In this paper Szilágyi and colleagues convincingly demonstrate that the origin of chromosomes must have preceded the origin of efficient specialist enzymes.

Such an important conclusion can only be reached by the rigorous numerical simulations (and not by speculation alone) that characterize the work of Szathmáry’s group.

The paper is clearly written, and it is shown that the conclusions are robust to changes in the parameters. I have a few comments that I hope the authors can address in a revised version.

First, Szilágyi and colleagues consider only the two extremes of linkage (all or none), which leaves open the question if more specialized enzymes could have been maintained by limited linkage. One can imagine that initially it was only pairs or small numbers of RNA genes that were linked. Would a cell with 2-gene chromosomes be able to outcompete a cell with no linkage and a cell with 3-gene chromosomes a cell with 2-gene chromosomes (and so on)? Demonstrating such graduality in the origin of chromosomes could provide a further valuable aspect to the model.

*We have included another version of the model that represents a transitional state connecting the fully independently replicating genes and the fully formed chromosome with controlled segregation (see revisedMethodsection). We demonstrate that linkage can go to fixation and linkage of genes in a chromosome is enough for full specialization, even without controlled segregation. Incidentally, this echoes the 1993 model of Maynard Smith and Szathmáry that did not model enzyme evolution, however. We are very grateful for the comment and we hope we were able to demonstrate that the transition from one system to the next is also possible.*

Second, the authors may consider discussing the issue of how the origin of efficient replicators relates to the origin of linkage. Since replication of chromosomes also requires efficient specialist enzymes (e.g. a primase, a replicase and a helicase), their origin must have also been influenced by assortment load. If the efficient replication of longer chromosomes requires multiple specialist enzymes, that can only evolve once chromosomes have appeared, this presents another error threshold-type problem.

*We agree that an error-threshold-like problem unfolds with independently replicating genes, apart from the one stemming from the mutational load: random assortment causes loss of genes, which can be tolerated to certain extent*[20]*, but limits the number of genes that can coexist. However, replicating a chromosome or individual genes requires the same set of enzymes. In essence, in the unlinked system there are as many chromosomes as there are genes (and each chromosome can be in multiple copies). Thus if we assume that the system with individually replicating genes can exist (which is an interesting question in its own right!), then the one with linkage does not need significantly more enzymes (see our discussion).*

In the discussion the authors write that “The fidelity of the replication process as well as the control mechanisms that guide the segregation of the chromosome evolved at this stage of the origin of life. Diversified, complex metabolism evolved afterwards”. Given that the fidelity of replication and the control mechanisms that guide chromosome segregation presumable also depended on specialist enzymes, I am wondering if all these properties may have rather coevolved with linkage.

*We agree that the complexity of metabolism coevolved with the genetic representation. Our results do not imply that no specific enzyme could evolve, only that given the possibility of generalist enzymes, evolution will not opt for them. Furthermore, a good replicase is a generalist enzyme, as it should take many kinds of substrate (different sequences) and copy them. At the same time it should also be an efficient enzyme, as it should work with high fidelity in replication. Higher efficiency, measured as flux, evolves in the simpler system as well* (Figure 6)*.*

The above considerations boil down to the question: rather than taking linkage as given, could selection for more efficient enzymes have driven the gradual origin of linkage?

We now demonstrate that, given the possibility of linkage, the higher efficiency attainable drives the system toward the fixation of chromosomes and in turn to full specialization.

## Appendix

### Calculation of the flux

Enzyme–substrate interaction. We assume the enzyme active sites as cubic cavities of *D* dimensions; substrates are *D* dimensional cubes and the activated complex as a cube centered in the cavity (with parallel faces). The interaction acts between the 2*D* pieces of *D*-1 dimensional faces of the cavity and the cube. Thus the steric interaction energy of the enzyme–substrate complex can be calculated by the van der Waals formula:

$$\varepsilon =2\sum_{j=1}^{D}\left(\frac{A}{R_{j}^{12}}-\frac{B}{R_{j}^{6}}\right)\text{,}$$

where *R*_*j*_ is the distance between the *j*th inner wall of the enzyme and the corresponding face of the substrate (see Figure 1b). In our simulations, following Kacser and Beeby [17] we used *A*=1 and *B*=10.

The catalytic activity of an enzyme depends only on the van der Waals interaction between enzyme and substrate and can be approximated as the logarithm of this energy.

$$e=\ln\left(-\varepsilon \right)\text{,}$$

where we fixed the proportionality to one. In our simulations to speed up the convergence we used simple proportionality between interaction energy and catalytic activity:

e=−ε.

We believe that this simplification is not qualitatively significant for our results. In case of non perfect functional group matching (i.e. waste catalytic directions) we assume a 0.5 penalization factor. Note that the optimal catalytic activity (which is independent of the size of the substrate) is:

$$e*=\frac{B^{2}}{2A}D\text{.}$$

Flux of a branched chain of reactions with unsaturated enzymes. Let us assume an *L*-step chain of reaction with waste products, see Figure 1 for *L*=*5*. Each reaction step (both forward and waste) is treated as a simplified Michaelis–Menten step with unsaturated enzymes. In this case the flux of an elementary reaction step is

$$I_{\mathrm{elem}}=e\left(\left[S\right]-\left[P\right]\right)\text{,}$$

where [*S*] and [*P*] is the substrate and product concentration, respectively. To get the final flux *I*_*L*_ we should introduce some more simplifications. We assume a large, well-mixed reactor and the waste concentration is treated as zero: [W_i_] ≈ 0. If one holds the initial and final substrate concentrations constant ([X_0_] = 1, [X_final_] = 0) we obtain the following linear system for the simplified kinetics

$$\begin{array}{l} I_{i}=e_{i}(\left[X_{i-1}\right]-\left[X_{i}\right]),\;(i=1,2,\ldots ,L)\\ {\tilde{I}}_{i}=e{'}_{i}\left[X_{i-1}\right],\;(i=1,2,\ldots ,L) \end{array}$$

for the waste and intermediate fluxes, and

$$I_{i}=I_{i+1}+{\tilde{I}}_{i+1}\begin{array}{cc} & \left(i=1,2\ldots L-1\right) \end{array}$$

for the flux conservation. The final flux *I*_final_ (i.e. the fitness) can be easily computed from this set of equations.

For *L=3* the set of equations defines the direct and waste fluxes:

$$\begin{array}{l} I_{1}=e_{1}(1-\left[X_{1}\right])\\ {\tilde{I}}_{1}={\tilde{e}}_{1}1\\ I_{2}=e_{2}(\left[X_{1}\right]-\left[X_{2}\right])\\ {\tilde{I}}_{2}={\tilde{e}}_{2}\left[X_{1}\right]\\ I_{3}=e_{3}(\left[X_{2}\right]-0)\\ {\tilde{I}}_{3}={\tilde{e}}_{3}\left[X_{2}\right]\\ I_{1}=I_{2}+{\tilde{I}}_{2}\\ I_{2}=I_{3}+{\tilde{I}}_{3} \end{array}$$

and the final flux *I*_*3*_ is the following:

$$I_{3}=\frac{e_{1}e_{2}e_{3}}{\left(e_{1}+e_{2}+{\tilde{e}}_{2}\right)\left(e_{3}+{\tilde{e}}_{3}\right)+e_{2}\left(e_{1}+{\tilde{e}}_{2}\right)}$$

An important special case is when all direct catalytic activities have the same value (e.g. their common maximal value e*, see previous section) and the waste activities are all zero. In this case the total flux (the theoretical maximum of the flux) is the following

$$I_{L}^{*}=\frac{e*}{L}=\frac{1}{L}\frac{B^{2}}{2A}D\text{.}$$

If the number of reaction steps is equal to the dimension of enzymes the optimal flux depends on van der Waals parameters only:

$$I_{L}^{*}=\frac{B^{2}}{2A}\text{.}$$
